# “I definitely feel like a scientist”: Exploring science identity trajectories among Latinx students in a critical race theory-informed undergraduate research experience

**DOI:** 10.1002/icd.2371

**Published:** 2023-02-02

**Authors:** Yolanda Vasquez-Salgado, Tissyana C. Camacho, Isabel López, Gabriela Chavira, Carrie L. Saetermoe, Crist Khachikian

**Affiliations:** 1 Department of Psychology, California State University, Northridge, California, USA; 2 Department of Child and Adolescent Development, California State University, Northridge, California, USA; 3 Department of Counseling, Clinical, and School Psychology, University of California, Santa Barbara, California, USA; 4 Department of Civil Engineering and Construction Management, California State University, Northridge, California, USA

**Keywords:** critical race theory, culture, Latinx students, science identity, undergraduate research experience

## Abstract

The current study investigated science identity development among Latinx university students selected for a critical race theory (CRT)-informed undergraduate research experience. Twenty students (12 female, 8 male; *M*_age_ = 22.00; *SD* = 2.77) enrolled in biomedical-related majors at a 4-year university responded to open-ended questions regarding their identity as scientists at 2 weeks, 6 months, and 18 months after they began the program. Results illustrated a steady increase in the number of students identifying as scientists over 18 months. At 2 weeks into the program, only 35% of Latinx students felt like a scientist. At 6 months, 45% of Latinx students identified as a scientist. At 18 months, 70% of Latinx students reported feeling like a scientist. Results also revealed variation in science identity trajectories, with four trajectories viewed in the data: (1) *consistent or fast achievement*, (2) *gradual achievement*, (3) *achievement adjustment*, and (4) *never reach achievement*. The majority of students demonstrated a trajectory in which they reached science identity achievement (the feeling that they are “a scientist”). Our results provide evidence of the positive, longitudinal impact that a CRT-informed curriculum has on the science identity development of Latinx students. Implications surrounding future research and strategies to facilitate long-term Latinx student participation in the biomedical sciences are discussed.

## 1 | INTRODUCTION

Recruiting, training, and retaining a diverse science, technology, engineering, mathematics, and medicine (STEMM) workforce has been a long-standing goal of the scientific community. While most work in STEMM has focused on striving for racial-ethnic diversity, in this paper, we refer to diversity that permits a balanced representation (Graham, 2018) across a range of sociocultural identities within the field of science (e.g., racial-ethnic, gender, socioeconomic). This has resulted in an advent of undergraduate research experiences (URE) and scholarships for students traditionally deemed as underrepresented minorities (e.g., African-American, Latinx), which we refer to as *minoritized* in this paper (acknowledging the power dynamics of such language; Ajayi et al., 2021). Although minoritized groups continue to increase numerically in the biomedical sciences, racial-ethnic disparities persist in educational and career outcomes (Funk et al., 2021; NCES, 2017). Resources provided by traditional UREs have certainly been helpful; yet, continuing to train and socialize minoritized students in the context of white, cultural norms is unlikely to yield greater racial-ethnic inclusion in the sciences (Camacho & Echelbarger, 2021). Thus, a URE must focus on developing and delivering a culturally affirming curriculum that recruits and retains emerging scientists whose sociocultural identities are acknowledged and connected with their engagement in science (Camacho et al., 2021). The current research focuses on exploring the science identity trajectories of Latinx students who engaged in such a curriculum through their participation in an intensive research program designed to diversify the biomedical research workforce over the span of 18 months.

### 1.1 | National statistics regarding STEMM and the Latinx population

According to the U.S. Bureau of Labor Statistics (2017), between 2009 and 2015, employment in STEMM-related fields grew by 10.5%, compared with 5.2% growth in non-STEMM occupations. STEMM-related employment is projected to further grow by 13%, compared with a 9% growth in non-STEMM fields between 2017 and 2027 (T. D. Jakes Foundation, 2020). Yet, individuals from Latinx backgrounds are less likely to pursue a STEMM education (National Science Foundation, 2021) than White and Asian-descent students, blocking them from these growing employment opportunities. Latinx individuals comprise 19% of the U.S. population aged 18–64, yet they earn only 14% of all STEMM bachelor’s degrees awarded and account for an even lesser share (9.6%) of the STEMM workforce. To address these educational and economic disparities, institutions must reflect on how they contribute to such inequities and consider innovative ways to reduce and eradicate these disparities.

To increase the participation and inclusion of Latinx individuals in STEMM, we must address that the status-quo of scientific training is falling short for minoritized students. Beyond the typical resources provided by traditional UREs, Latinx students can benefit from a curriculum that reflects their lived experiences, rooted in culturally relevant and empowering pedagogy (Camacho & Echelbarger, 2021; Pak, 2018). By practicing such a pedagogy through an empirical basis, researchers can come to understand the processes involved in the development of Latinx students’ commitment, persistence, and eventual pursuit of a science career. One key component associated with one’s commitment, persistence, and eventual pursuit of a science career is *science identity* (e.g., Chang et al., 2011; Chemers et al., 2011; Espinosa, 2011; Estrada et al., 2011). Thus, to better understand how to promote scientific career pursuits among Latinx students, further investigation of how Latinx students come to their science identities is warranted.

### 1.2 | Science identity and the Latinx population

Science identity is broadly understood to reflect one’s personal belief that they are a scientist (Thompson & Jensen-Ryan, 2018) as well as the perception by others that they are indeed a part of the scientific community (Carlone & Johnson, 2007). Science identity is built through a dynamic, multidimensional process that develops over time and is associated with one’s ever-changing social experiences rather than a static state of being (Erikson, 1968; Arnett, 2000). That is, identity is something that is developed over time, rather than an innate attribute that one simply has or does not have. According to Brickhouse and Potter (2001), by focusing on the concept of identity, we can draw attention to the individual (e.g., agency), and at the same time, expand our view of the individual to include their construction of the social structures that may influence them. Thus, in investigating science identity, one must also consider the strengths and barriers that play a role in forming one’s personal sense that they are a scientist.

Investigating the science identity of minoritized students, such as those from Latinx backgrounds, is of particular importance given the increased likelihood of social barriers that they face in pursuit of a science degree and career (Chang et al., 2014; Lu, 2015) as well as social barriers that disrupt college degree attainment for minoritized students in general (Solórzano et al., 2005). Compared to white/non-minoritized students in STEMM, minoritized students are more likely to be first-generation college students (Bettencourt et al., 2020; Vasquez-Salgado et al., 2021), less likely to have social support (Chang et al., 2014), and less likely to feel a sense of belonging (Hurtado et al., 2007; Oldfield, 2007). They are also more likely to switch out (or be pushed out) of STEMM majors (Baird et al., 2016; Chang et al., 2014). These factors likely contribute to Latinx students experiencing difficulties in viewing themselves as scientists. However, minoritized students who consider science as an important aspect of their identity are more likely to persist in a STEMM major (Chang et al., 2011; Espinosa, 2011), underscoring the central importance to delve deeper into the experiences that promote science identity of Latinx undergraduate students.

### 1.3 | Science identity development in college

To understand how Latinx students come to view themselves as scientists (i.e., their identity development), we must examine how the concept of science identity changes over time among Latinx students. A predominant model of identity formation states that the development of one’s social identity can be categorized into several statuses: three statuses (e.g., Phinney, 1989; Syed et al., 2007), four statuses (e.g., Marcia, 1966), or five statuses (e.g., Crocetti et al., 2008). We sought parsimony—aiming for the simplest measurement of a concept; therefore, the current study considers a three-status approach that includes an *achievement* status (i.e., having explored and internalized one’s identity), a *moratorium* status (i.e., having explored but not yet internalized one’s identity), and an *unexamined*^1^ status (i.e., minimal exploration and no clear internalization of one’s identity). Research with different domains of social identity have noted that there is typically a developmental progression towards identity achievement, especially throughout the college years (Meeus et al., 1999; Seaton et al., 2006). Identity achievement is a primary developmental goal of emerging adults enrolled in college (Arnett, 2000; Arnett, 2014), as a myriad of work demonstrates that such “achieved” social identities are beneficial to the students’ psychological health and academic outcomes (e.g., Rivas-Drake et al., 2014).

Despite the notion of “identity achievement,” research demonstrates that even college students with an “achieved” identity at one time may shift into a status of moratorium or unexamined identity, depending on their lived experiences and changes in environment (Syed et al., 2007). Thus, it is important for researchers to capture students’ perceptions of themselves as scientists across time and move beyond investigating such perceptions at a single point in time. Investigating how and why science identity changes over time will help explain identity patterns, as well as what promotes (or hinders) such identity development—an important step to creating an inclusive science.

Although information about the characterizations of other domains of identity (e.g., gender, ethnic identity) has enabled us to understand the concept of identity in general, there is a pressing need for more research on how science identity *changes over time* among college students. Robinson et al. (2018) investigated science identity trajectories (changes in identity over at least three periods of time) of science majors at an elite university across their 4 years of enrolment at the institution, measured as the importance of science to participants’ identity. The findings revealed three patterns of science identity throughout college among a multi-ethnic sample: (1) students who began college with high levels of science identity and remained relatively high, (2) students who began college with moderately high levels of science identity and remained at such levels over time, and (3) students who began college with moderately low levels of science identity and sharply declined over time. Additionally, Robinson et al. (2018) found that minoritized students were more likely to belong to the science trajectory group characterized by moderately low levels of science identity (with a sharp decline) throughout the college years, a finding that was also displayed when examining changes in science identity over a semester (Robinson et al., 2019). The researchers argued that science identity can be strengthened by institutionalized support, such as involvement in UREs. Indeed, prior research shows that undergraduate Latinx STEMM majors who participated in a culturally informed URE reported higher levels of science identity and a commitment to pursue science than matched Latinx STEMM peers who did not participate in the culturally informed URE (Camacho et al., 2021). This finding suggests that an institutional intervention can influence science identity among Latinx college students. Similarly, McCartney et al. (2022) found an increase in science identity in biology undergraduate students through a semester-long professional development intervention. The current study extends prior research by examining the qualitative ways in which science identity changes over time for Latinx students when they receive institutional support via involvement in a culturally affirming URE.

### 1.4 | BUILD PODER: A critical race theory-informed undergraduate research experience

Funded by a grant from the National Institutes of Health, Building Infrastructure Leading to Diversity (BUILD), Promoting Opportunities for Diversity in Education and Research (PODER), at California State University, Northridge (CSUN), is a URE founded in critical race theory (CRT; Solórzano et al., 2000) to increase the representation and persistence of minoritized students pursuing biomedical science careers (Saetermoe et al., 2017; Vargas et al., 2021). The program offers traditional components of UREs (e.g., apprentice-style research laboratory participation under faculty advisement, required presentation of research findings); however, students experience scientific inquiry in collaboration with faculty mentors who are trained in culturally responsive mentorship (Vargas et al., 2021) while students conduct research on health disparities or research from the lens of social justice. Faculty mentors are trained to teach students to “navigate the cultures and discourses of STEM fields [and] enter and interact successfully in professional STEM networks” (Bensimon & Dowd, 2012, p. 3). While the URE program staff are ethnically diverse, and the program directors are both of Latinx descent, STEMM students are unlikely to be ethnically/racially matched with their faculty mentors. While Latinx students at CSUN comprise 56% of STEMM majors, there are fewer than 8% of Latinx faculty in STEMM fields (CSUN, Institutional Research, 2022). We find it essential that student mentees and faculty mentors (individually and collaboratively) are trained in the central tenants of CRT in education (Solórzano & Yosso, 2002) through a host of activities (for more information, see Table 1 in Camacho et al., 2021).

#### 1.4.1 | Selection of BUILD PODER students

BUILD PODER selects undergraduate students through a holistic consideration and review of seven criterion: (1) currently enrolled undergraduate at CSUN (the institutional location of the program) or incoming community college transfer student, (2) full-time enrolment status, (3) a major relevant to biomedical sciences (e.g., public health, biology), (4) grade point average at or above 3.0, (5) U.S. citizen or noncitizen nationals or permanent residents, (6) identification with a historically minoritized group (e.g., ethnic-racial, gender identity, and sexual orientation), disability, or economically disadvantaged background, and (7) a 2-year commitment to be in the program (at minimum), with completion of at least 30 units of college-level courses. As a federally funded program, one of the greatest challenges to equitable access for all students was the issue of citizenship. Eventually, we were able to secure some institutional funds for a limited number of undocumented students.

#### 1.4.2 | General curriculum

BUILD PODER’s CRT curriculum makes both students and their faculty mentors aware of power structures within the scientific community. But awareness of structural inequities, such as racism, is insufficient. We harness the power in students’ *experiential knowledge* or lived experiences, a key CRT tenant, and their community cultural wealth (CCW, Yosso, 2005), a complementary view, to empower students by making them realize their strength in overcoming those systems, as a collective force, both now and in the future. We strive to provide an environment for students to not only *survive*, but *thrive* in the sciences through workshops, mentorship, and a community of support that students can always rely on when faced with issues of adversity. We serve as a community or “homebase” for students—a place they can always turn to for support and guidance. A recent study shows that graduating seniors in the URE were more likely to persist and pursue science careers compared to science-active and mentored students not in the URE (Camacho et al., 2021). Together, CRT and CCW challenge traditional views of capital and use asset-based approaches that recognize the “cultural knowledge, skills, abilities, and contacts” of minoritized groups that are unacknowledged (Yosso, 2005). Throughout the student training, which takes place across two consecutive academic years (including two summers), the curriculum challenges traditional stereotypes and beliefs about who a scientist is through several avenues (via curriculum informed by the tenants of CRT in education; Solórzano et al., 2000). The curriculum also empowers students by guiding them in recognizing the vast cultural wealth they bring from their families and communities.

Upon acceptance into the program, students enter an intensive, full-time, 4-week JumpStart program with their cohort. As a cohort, students work collaboratively in research-related activities to develop a community and culture of support while being provided on-campus housing and three meals a day. JumpStart activities challenge traditional research training programs by focusing on the *importance* of culture and various forms diversity (e.g., ethnic, gender, socioeconomic) within the scientific community. Additionally, the *benefits* to the science community of incorporating diverse perspectives and beliefs are discussed, and students come to recognize that their experiential knowledge is recognized and valued in the program (Yosso, 2005). For example, in JumpStart, students interview family members who are the holders of knowledge (i.e., linguistic, values, traditions) in their communities who may use non-Western forms of healing or other forms of knowledge that may have informed students’ scientific curiosities or sparked their interests to contribute to science and their own communities.

During the academic year, students work in a faculty mentored biomedical research laboratory, take advanced research methods and professional development courses, and participate in biweekly group-based discourse and activities that create a sense of belonging in the sciences. BUILD PODER therefore provides the traditional elements of many UREs, but intentionally encompasses a culturally embedded curriculum founded in CRT. Such an approach reflects that diverse student groups bring with them rich prior experiences and knowledge about science, as well as their own ways of knowing, thinking, and communicating; these experiences and skills are fruitful to their learning in educational settings (Bryan & McLaughlin, 2005; Lemke, 2001; Wall, 2016; Yosso, 2005).

Though prior UREs have conducted exploratory analyses on minoritized students’ science identities (e.g., Hurtado et al., 2009), few have explored the developmental paths or trajectories of students’ science identities over the course of their participation in the URE. Moreover, to our knowledge, no UREs have intentionally developed their curriculum through a culturally affirming lens such as CRT. Consequently, given the dearth in the literature and unique nature of BUILD PODER, the current paper includes an in-depth analysis of Latinx students’ science identity trajectories (i.e., changes in science identity over time) that participated in the program.

### 1.5 | Gender and science identity

Researchers examining science identity among students in STEMM and STEMM-related fields have noted some differences between male and female students. In a number of studies, females tend to demonstrate lower rates of endorsing a scientist identity compared to males (Cundiff et al., 2013; Hazari et al., 2013). These rates mirror the nationwide rates where there is a lower percentage of females compared to males in science careers (NSF, 2017). For example, in an assessment of students’ self-evaluations across science fields of study (i.e., biology, chemistry, and physics), significant gender differences emerged between male and female students’ evaluation of themselves as scientists in the field of physics, with female students reporting a lower level of science identity compared to male students (Hazari et al., 2013). In contrast, there were no significant differences between males and females in the fields of biology and chemistry. Moreover, Latinx females reported significantly weaker science identities compared to Black and White females across all science fields (Hazari et al., 2013). Thus, though gender differences were present, they are amplified in Latina youth who are potentially more at risk for weaker science identity and, hence, lower pursuit of a science career.

Given such findings, researchers have been compelled to delve into why female students do not typically demonstrate comparable rates of endorsing a science identity compared to male students. A major area of study includes examining the impact of science stereotypes; such stereotypes can give rise to stereotype threat, which impact confidence, anxiety, and communal utility value (i.e., the ability to serve community within the science field of study; Smith et al., 2015). Indeed, research has documented the impact of science stereotypes on the development of science identity and how this may impact female and male students’ identity and career aspiration as scientists (Cundiff et al., 2013). In a sample of college students taking science major courses, it was revealed that female students who reported a greater endorsement of stereotypes held weaker science identities and aspirations. In contrast, male students who adopted gender-science stereotypes demonstrated greater science identity and aspirations for a career in science (Cundiff et al., 2013). Science-related stereotypes invite male students and disinvite female students, reinforcing inequities. Considering the findings on science stereotypes and the role they play in Latinx female students’ science identities, URE programs like BUILD PODER—which seek to make students aware of and overcome the many science stereotypes that exists (e.g., ethnic, gender)–are critical for eliminating such gender disparities. Although prior cross-sectional and longitudinal research demonstrates that female students are less likely to report a strong science identity (Cundiff et al., 2013; Hazari et al., 2013; Robinson et al., 2018), we do not know whether gender differences in science trajectories will be present among Latinx students who participated in BUILD PODER, as it is a program that intends to honor and integrate the many and various sociocultural identities students hold. This is an important question that the current research addresses.

### 1.6 | Current study

The purpose of the current study was to explore the developmental nature of science identity over the span of 18-months for Latinx undergraduate students as they engaged in a CRT-informed URE, BUILD PODER. Specifically, utilizing an identity statuses framework (Marcia, 1966) we sought to test the impact of a CRT-informed curriculum on Latinx undergraduate students’ science identities. In doing so, we document their science identity statuses and longitudinal trajectories. We also investigate whether science identity trajectories vary by gender. Three research questions guide our investigation.

### 1.7 | Research questions

1. What type of science identity statuses do Latinx undergraduate students ascribe to during their involvement in the program (i.e., identity achievement, moratorium, unexamined)?
2. What patterns of change do Latinx students’ science identity trajectories take over the course of 18 months?
3. Are there gender differences in Latinx students’ science identity trajectories?

## 2 | METHOD

### 2.1 | Participants

Undergraduate students enrolled in biomedical-related majors (e.g., biology, biochemistry, psychology) in BUILD PODER at CSUN, as part of their training curriculum, responded to open-ended questions asking about their identity as scientists at 2 weeks (Wave 1), 6 months (Wave 2), and 18 months (Wave 3) post enrollment in the program. Responses from 44 students were obtained. However, eight students did not provide a response to at least one of the three waves, bringing the sample size down to 36. Because our study focused on Latinx students, this further brought our sample down to 20 (12 female and 8 male students). The 20 Latinx students who completed all three waves of data collection did not differ in any way (e.g., major, gender) from the Latinx participants who did not complete all of the waves. Students were enrolled as rising sophomores, juniors, or seniors during the onset of their involvement in the program, and about 45% had transferred from a 2-year community college to the 4-year university. All remained enrolled in the program and CSUN throughout data collection periods. Eighty percent (16 students) were first-generation college students, with neither parent having received a college degree, and 80% were eligible to receive federal financial assistance (i.e., Pell Grant).

### 2.2 | Science identity measures

Open-ended questions pertaining to students’ identities and experiences as scientists were asked across three separate waves. At Wave 1 (2 weeks post-enrollment), students were asked: “*Do you see yourself as a scientist? Why or why not?*” At Wave 2 (6 months post-enrollment), students were asked: “*Can you tell us how you are feeling about your skills as a researcher? Are you starting to feel like a scientist? Why or why not?*” At Wave 3 (18 months post-enrollment), students were asked: “*Can you tell us how you are feeling about your skills as a scientist? Do you feel like a scientist? Why or why not?*

While there was some variability in the wording of the open-ended questions that were distributed across each wave, all questions gauged students’ self-reported identity as scientists. Thus, the underlying notion of asking for their identity as scientists was consistent. Indeed, all questions were asked at the same point in time for all participants and centered on the same point of interest: whether they identified or felt like a scientist, and why or why not. Taken together, the open-ended, multiple-question format enabled us to understand whether students did or did not perceive themselves as scientists.

### 2.3 | Procedure

Students newly accepted into BUILD PODER were required to sign consent forms and to include their curriculum materials, including self-assessments, for formal evaluation of the program (all approved by the University’s Institutional Review Board). Students are formally assessed and evaluated throughout their 2 years in the program.

The first wave of assessment took place in-person (i.e., hand-written responses), and data for the second and third waves were collected via an online assessment. Wave 1 (2 weeks post-enrollment) hand-written responses to the open-ended questions were transcribed verbatim into an electronic format to compare the electronic data from Waves 2 (6 months post-enrollment) and 3 (18 months post-enrollment). The timing of assessments was determined by the BUILD PODER director and aimed to capture students’ science identity with just 2 weeks of exposure to the CRT-informed curriculum, and thereafter, half a year and 1.5 years into the program.

### 2.4 | Data analysis

Open-ended responses at each wave were analysed independently through a theory driven approach of *deductive* coding, relying on a theory to guide our initial coding (Braun & Clarke, 2006). We moved to a numeric analysis of qualitative data in order to examine science identity frequencies and trajectory trends. Thereafter, we enriched our quantitative arguments with quotes that illustrate narrative responses to each of our research questions.

#### 2.4.1 | Deductive coding

First, to examine identification as a scientist, a thematic analysis (Braun & Clarke, 2006) was conducted using a *deductive approach*. This analysis reflects a coding scheme that was based on existing theoretical and empirical work. Specifically, consistent with previous research examining change in identity using identity statuses (e.g., Syed et al., 2007), we coded science identity statuses as (1) *achieved (scientist)*, (2) *moratorium (unclear)*, and (3) *unexamined (not a scientist)*. It is important to mention that the term, “not a scientist”, was incorporated into the traditional “unexamined” identity status code in order to account for students that did not recognize themselves as scientists. This aligns with other models of science identity whereby an individuals’ science identity is dependent on whether or not they recognize themselves as a “science person” (Carlone & Johnson, 2007). Moreover, the coding scheme was created by the first author, who coded all responses and trained a graduate student (third author) to code the data independently. The interrater reliability for the science identity analysis was 0.70 for Wave 1, 1.00 for Wave 2, and 0.90 for Wave 3. The slightly lower interrater reliability in Wave 1 may be due to the initial novelty of the coding scheme for the coder that was trained. In the event that the first and third authors could not collectively decide on a specific code, suggestions from the second and fourth authors were solicited in facilitating a final code. The pattern of science identity codes across the three waves were thereafter examined for emergence of patterns in science identity trajectories by the first author (see Table 1 for full list of science identity themes and sub-themes).

#### 2.4.2 | Narrative responses

In order to maintain the confidentiality of participants, we incorporated students’ major in a broad fashion, and omitted instances where participants referred to names, specific individuals, or particular research institutions, from the manuscript. Where necessary, commas or semicolons were added to segment a narrative and make its meaning clearer. Narrative material between brackets means that it was added by a researcher when words were implicitly assumed or when something was inserted in order to maintain anonymity.

In reporting the narrative responses, the gender (female or male) of the participants and the wave (in some instances) were labeled. In order to ensure that identity statuses were clear for readers, irrelevant information was eliminated from a participant’s response; these omissions were denoted by an ellipsis. This style of narrative presentation has been used in prior qualitative research (Burgos-Cienfuegos et al., 2015; Vasquez-Salgado et al., 2015).

## 3 | RESULTS

### 3.1 | Science identity statuses

Our first research inquiry centered around exploring the types of science identity statuses (*achievement*, *moratorium*, and *unexamined*) that Latinx undergraduate students adhered to during their involvement in the BUILD PODER URE. In an examination of the responses across each wave (i.e., 2 weeks, 6 months, 18 months post-enrollment), participants varied and changed in their views of themselves as scientists, mirroring prior identity status research (e.g., Syed et al., 2007).

#### 3.1.1 | Achievement

Participants were classified into a science identity achievement status if they explicitly stated that they were scientists and did not mention any barrier in their pathway. If participants did not explicitly use the term ‘scientist’, but provided a response that indicated devotion or passion for research, they were classified into a science identity achievement status (this occurred in two instances). In Wave 1, seven students (35%) were coded as having achieved a science identity. In Wave 2, nine students (45%) were coded as having achieved a science identity, and in Wave 3, 14 students (70%) were coded as having achieved a science identity. A narrative illustration of an achieved identity status is provided below:

Yes, I definitely feel like a scientist. I am able to build a study from the ground up and apply my skills to help my lab mates, as well as open [the] door for myself. I’m so excited to be going to [Research Institution] for 8 weeks… [Female student, Wave 2]

#### 3.1.2 | Moratorium

Participants were classified into a moratorium status if they explicitly stated they viewed themselves as scientists but noted barriers (e.g., lack of skills or confidence) in their endeavours. We labeled this as moratorium because we postulated that such barriers prevented students from feeling secure in their roles as scientists. A lack of security meant that science identity was not fully achieved. Furthermore, participants who did not explicitly view themselves as a scientist but described themselves as developing (e.g., starting or beginning to feel like a scientist) or developing in the long term (e.g., will occur in the future) as a scientist were also classified in moratorium. These types of descriptors align with literature that recognizes moratorium as a general stage of identity exploration that may also include elements of uncertainty (Marcia, 1966; Phinney, 1989). Of the 20 students, six (30%) in Wave 1, nine (45%) in Wave 2, and four (20%) in Wave 3 were classified in moratorium. A narrative illustration of a moratorium identity status is provided below:

I am feeling somewhat confident in my skills as a researcher. I just need to complete some classes to learn the research methods that go with [major]. After this course, I will feel more confident on doing things on my own. So far, I lack the skills to help the graduate students in their projects. I understand that it takes time to learn and I should be more patient, but I feel useless when my colleagues are working so hard on their individual projects and writing abstracts for conferences. I want to help, but I can only sit back and write notes on what they are doing… [Male student, Wave 2]

#### 3.1.3 | Unexamined (not a scientist)

Participants were classified into an unexamined (not a scientist) science identity status if they explicitly stated that they did not think of themselves as scientists or if they never provided a response or indication regarding their thought processes surrounding their role as scientists. Of the 20 students, seven (35%) in Wave 1, two (10%) in Wave 2, and two (10%) in Wave 3 were classified as unexamined. It is noteworthy to mention that there was one participant who was classified into an unexamined science identity status across all waves. A narrative illustration for an unexamined (not a scientist) identity status is provided below:

As I am now, I fail to see myself remotely resemble a scientist. A scientist for me is a person who has published an article in an academic journal as a primary author. Until I have accomplished this task I will still feel as though I lack the ability to call myself a scientist. [Male student, Wave 1].

### 3.2 | Science identity trajectories

While participants’ science identity statuses were assessed across each wave, the nature of their science identity trajectory varied. That is, the status of science identity from 2 weeks to 18 months into the program varied over time within and across the participants. In examining the various trajectories that were demonstrated across the participants, four major trajectories were observed in the data: (1) *consistent or fast achievement*, (2) *gradual achievement*, (3) *achievement adjustment*, and (4) *never reach achievement*.

#### 3.2.1 | Consistent or fast achievement

Nine out of 20 participants (45%) fit a consistent or fast achievement trajectory pattern (see Table 2). Among those who fit this trajectory were participants who reported feeling like a scientist across all three waves (*n* = 4), or rapidly shifted from unexamined (*n* = 2) or moratorium (*n* = 2) science identity status in Wave 1 to achieved status in Wave 2, and remained in achieved status in Wave 3. It is also noteworthy to mention that one participant was in an achieved identity status in Waves 1 and 3, but in a moratorium status in Wave 2. Because this participant held science identity achievement statuses in two of the three waves and illustrated achievement in the final wave, they were placed in this trajectory group. Below is an example of one participant who illustrates this trajectory, as she held a science identity achievement status in all three waves:

#### 3.2.2 | Case: Female student (consistent or fast achievement)

Wave 1: When I think of my identity, as a scientist, I picture an ordinary young Mexican woman. I see myself standing tall, standing up for what I believe in, and taking control. Not letting anyone bring me down because I am not a male or white scientist. I want to be a scientist that motivates other young women that look just like me to make them believe that they can do it too.

Wave 2: For my first [semester] as a research assistant, I feel like I gained a lot of skills. I learned to work with others due to the various lab members and I also learned how to speak more professionally to faculty. I would’ve never imagined myself doing research and now I cannot imagine my life without it.

Wave 3: I do feel like a scientist. Through the help of BUILD PODER and my mentor, I have gained many professional skills that makes me feel like a scientist. For example, one thing that makes me feel like a scientist is having the ability to present my research at professional conferences.

In the case above, the participant identified as a scientist at Wave 1. She continued with this identification at Wave 2, noting her research experiences and that she could not imagine her life without research (indicative of devotion and passion for science). Finally, the participant maintained her achievement status by stating that she felt like a scientist at Wave 3. Although her language throughout each of the waves slightly varies, the premise is the same: the participant is connected to the view that she is a scientist.

#### 3.2.3 | Gradual achievement

Five out of 20 (25%) participants fit a progressive trajectory whereby science identity achievement was eventually reached at Wave 3 (see Table 2). This also implied that in earlier waves, participants were either unexamined or in moratorium regarding their identities as scientists. Below is an illustration of a gradual science identity achievement trajectory:

#### 3.2.4 Case: male student (gradual achievement)

Wave 1: I do believe that one day I can become a scientist but currently I do not feel like I am ready be called one. There are many definitions of a scientist but my definition entails working in a lab, making discoveries and publishing those discoveries and since I have not worked in a lab or have not published anything, I do not consider myself to be a scientist…

Wave 2: I am starting to feel like a scientist, but I still feel like I need to learn more skills and obtain more experience. Having more than one mentor can help.

Wave 3: I feel like I have discovered little by little throughout my time at BUILD PODER that I am capable of becoming the scientist I always imagined I could be. Today I do feel like I have become that scientist…

In this example, the participant began in the unexamined status (not a scientist) at Wave 1, progressed to the moratorium status at Wave 2 (as he still experienced some barriers), and eventually reported feeling like a full-fledged scientist at Wave 3. It is interesting that his own words, “little by little”, encapsulates the essence of his science identity trajectory. Though, initially, he did not see himself as a scientist, he foresaw that he could become one and he indeed, became a scientist via a trajectory that mimicked quantitative-like shifts in development.

#### 3.2.5 | Achievement adjustment

Two of 20 participants (10%) fit an achievement adjustment trajectory pattern (see Table 2). That is, they were classified as an achieved identity status at some point, but at Wave 3, adjusted their self-perceptions to a moratorium status. An achievement adjustment trajectory may reflect a participant’s sense of reevaluating their identities as scientists in light of their thought processes or experiences over time.

#### 3.2.6 | Case: male student (achievement adjustment)

Wave 1: I think I have viewed myself as a scientist even since I was a child because I always had to use what I had, which was not very much. I would make/build things that I thought was pretty cool such as water balloon traps, sling shots, and pretty much everything I have seen or could think of. I also feel like a scientist because I eventually try to recreate experiments I find interesting such as the effect of Boyle’s law at the dorm pool. As a scientist, I try to gain all the knowledge I can and frequently work to continue to improve myself. I do not like; however, the fact that when people say “picture a scientist”, they immediately picture a white man in a lab coat. I have seen countless other people, all with different attractions, who are excellent scientist, but still do not get recognized by society. I myself do not fit the stereotype, except for being a male, but do see myself as an excellent scientist who will succeed in the field of [major].

Wave 2: Yes, definitely!

Wave 3: I feel like I do not know how to put the pieces together when it comes to viewing individual research papers and applying it to my work. This is most likely a result of not having to independently search for papers as my project is fairly straight forward. I actually feel somewhat discouraged as a scientist because I failed to do this in my summer research program and was called out on it. I think it would be greatly beneficial if there were a weekly journal club where we can discuss scientific papers within our respective fields with others.

In this example, the participant initially was in an achieved identity status at Waves 1 and 2. However, at Wave 3, he was in a moratorium status; though he still referred to himself as a scientist, he noted barriers he experienced in his external summer research at a doctoral granting institution that discouraged him. This trajectory reflects an adjustment or reevaluation that may be necessary for students in order to become aware of their shortcomings and make plans to gain skills that they lack. Once they acquire these skills, they may feel more confident in themselves as scientists.

#### 3.2.7 | Never reach achievement

Four out of 20 participants (20%) demonstrated a trajectory pattern that never reached an identity achievement status (see Table 2). This included participants who remained consistently in an unexamined status or consistently in a moratorium status. It also included participants who shifted between unexamined and moratorium statuses, but never reached an achieved science identity status. In such cases, participants never explicitly or implicitly (via their devotion, passion or excitement for research) stated that they were scientists. Below is an illustration of a participant that never reached an identity achievement status:

#### 3.2.8 | Case: female (never reach achievement)

Wave 1: I think that if I work really hard and stay motivated and driven that I can be a scientist and achieve my goals. I used to not see myself as a scientist because I felt like I did not look serious enough and that people would not take me serious because of how I look. I used to wonder how people would trust that I am capable with how I look/sound. Now I barely see myself as a scientist but I wish I had more skills and things research related that I am capable of doing well. I think that I can be a scientist if I had more skills and if nothing else happens that might prevent me from becoming a good scientist. I’m a scientist because I’m capable of thinking and doing research even though I can barely do anything related to science well. In my mind a scientist wears a white lab coat and does a lot of research and has a lot of sleepless nights. This is just a stereotype because not all scientists wear lab coats all the time. It does not matter what someone looks like on the outside because a scientist can look like any type of person.

Wave 2: I am starting to feel like a scientist because I feel like I am extremely busy like some of the scientists that I admire and look up to for all they are able to do. I am not too confident in my skills as a researcher as I still feel very lost on what to do. I wish I had more experience and that I had been more useful in my mentor’s lab to get more research experience…

Wave 3: I do not feel like a scientist, I feel unprepared for graduate school.

At Wave 1, the participant noted a foreshadow of the possibility of something preventing them from becoming a scientist. At Wave 2, her internal barriers became lived and explicit, and ultimately, at Wave 3, she submerged from a moratorium status to an unexamined status (i.e., does not see herself as a scientist). It is possible that her thoughts surrounding graduate school preparation triggered stereotype threat or social comparisons, as prior research has indicated that such comparisons are typical in science-based programs (Hurtado et al., 2009).

### 3.3 Gender differences in science identity trajectories

The final aspect of our research inquiries centered on examining whether there were gender differences in Latinx students’ science identity trajectories. In looking at the different science identity trajectories, we found evidence for gender differences (see Table 2). In the consistent or fast achievement trajectory group, all nine (100%) identified as female. In contrast, in the gradual achievement trajectory group, 4 of 5 (80%) identified as male, and both students in the achievement adjustment trajectory group identified as male as well. However, there was an equal number of males (50%) and females (50%) who never reached achievement. Moreover, to test whether females were more likely to hold a consistent or fast achievement science identity trajectory, and males a gradual achievement or achievement adjustment science identity trajectory, a Fisher’s exact test was conducted, and it was significant, *p* = 0.001. Together, these findings signify emerging evidence that gender might play a role in students’ science identity trajectories, with females being more likely to be consistent or fast achievers, and males being gradual achievers or achievement adjusters. It is important to mention that though we found gender to play a discernible role in Latinx students’ science identity trajectories, there did not appear to be any noticeable differences across major or education level at program entry (see Table 2).

## 4 | DISCUSSION

The current study provided an in-depth, examination of science identity among 20 Latinx students participating in BUILD PODER, a critical race theory (CRT)-informed URE designed to provide science experiences while uplifting minoritized students’ sociocultural identities. We found that over the course of 18 months, Latinx student participants varied in their levels of identifying as a scientist and varied in their processes of coming to feel like a scientist, with most identifying as a scientist at the final wave of investigation. Our results provide evidence of the positive, longitudinal impact that a CRT-informed curriculum has on the science identity development of Latinx students. They also highlight how transformative the time in college is for identity development, and how institutions can capitalize on such identity development to ensure academic success of minoritized students pursuing science (Ajayi et al., 2021; Camacho & Echelbarger, 2021).

### 4.1 | Science identity statuses

Like other research analysing identity statuses (e.g., Meesus et al., 2012; Phinney, 1989; Syed et al., 2007), we found that our sample demonstrated characteristics of several identity statuses and that identity statuses changed over time. Over each wave, a steady increase was observed in the percentage of students who identified as a scientist. Two weeks into their participation, only 35% of Latinx students felt like a scientist. An increase was seen 6 months into BUILD PODER where 45% of the students identified as a scientist, and at 18 months into the program, 70% of participants reported feeling like a scientist. Although this analysis does not consider within-person variation, it demonstrates that—as a whole—the program is having a positive impact on science identity development for Latinx students.

Within a year-and-a-half, the majority of students (70%) were categorized as having achieved an identity as a scientist and only two participants were categorized as *unexamined (not a scientist)*. The concept of “*not a scientist*” was incorporated into the traditional “unexamined” identity status in order to account for students that did not recognize themselves as scientists. Thus, it is not that they have *not* examined their science identity. These students stated their perceptions of a scientist did not align with what they felt they could offer (e.g., publishing a manuscript). It seems that some of the traditional perceptions of being a scientist are difficult to dismantle, and there are individuals who may need more time and resources to reimagine their schema of a scientist. Nonetheless, the majority of students do feel that they are a scientist and provided positive perceptions of their competency and confidence.

### 4.2 | Science identity trajectories

In our investigation, we also used students’ open-ended responses to investigate if discernible, within-person variation trends existed in Latinx students’ science identity over time. Four science identity trajectory patterns were observed in the data: (1) students who consistently felt that they were a scientist, or quickly came to feel that they were a scientist (i.e., *consistent or fast achievement*); (2) students who eventually identified as a scientist (i.e., *gradual achievement*); (3) students who reported feeling like a scientist, but eventually retreated from those statements (i.e., *achievement adjustment*); and (4) students who consistently did not feel like they were a scientist (i.e., *never reach achievement)*.

Similar to previous research on science identity trajectories, we found that there are several patterns that characterize how Latinx students can identify as a scientist over time (Robinson et al., 2018; Robinson et al., 2019). Previous research suggests that minoritized students, such as Latinx students, are more likely to be in trajectory groups that reflect lower levels of science identity (Robinson et al., 2018; Robinson et al., 2019), and postulated that perhaps these low levels of science identity could be altered or strengthened to higher levels of science identity with greater institutional support. Indeed, our findings demonstrate this to be the case. Among our sample, 70% of the Latinx students demonstrated a trajectory pattern in which they reached science identity achievement—the feeling that they are a “scientist” — within 18-months suggesting that Latinx students may identify as scientists when a URE takes a culturally affirming approach. These findings illuminate the importance of continued institutional support for programs, such as BUILD PODER in serving as mechanisms for promoting science identity (Camacho et al., 2021). Our findings align with our recent work that demonstrated Latinx STEMM students in BUILD PODER, a culturally affirming URE, had significantly higher levels of science identities and were more committed to pursuing a science-related career than similar STEMM students not in the URE (Camacho et al., 2021).

Identifying how one’s science identity develops over time is an important goal-worthy pursuit in higher education, as it provides us with an understanding of the foundational pattern of science identity development. While prior research has investigated the process of science identity development through quantitative measures (e.g., Robinson et al., 2018; Robinson et al., 2019), our study examined the perceptions of science identity through the written reflections of our participants. One way in which minoritized scientists are deemed less than is through *epistemic exclusion*: the devaluing of different ways of knowing and learning (Settles et al., 2020). The current study broadens our understanding of science identity development among minoritized groups, such as Latinxs, and demonstrates that deficit narratives that are predominantly used for minoritized students can be counteracted when taking a culturally affirming, strengths-based approach to their science learning and development in higher education.

### 4.3 | Gender differences in science identity trajectories

Finally, this study examined whether there were gender differences in developing a science identity, given the troubling trends where Latinx females report the lowest levels of feeling like a scientist (Hazari et al., 2013). Our study shows that Latinx females demonstrate science identity trajectories characterized by higher levels of science identity. In fact, all the students in the *consistent or fast achievement* trajectory (consistently felt that they were a scientist, or quickly came to feel that they were a scientist) identified as female. Thus, our findings suggest that Latinx females may be particularly benefitting from participating in a URE program like BUILD PODER that emphasizes CRT. Although males also benefitted from the program, their science identity trajectories appeared to be mixed: most fit a *gradual achievement* (eventually identifying as scientists by 18 months) or *achievement adjustment* (initially reported feeling like a scientist, but eventually retreated from those statements) trajectory. However, it is important to note that this may also be due to other factors, such as the fact that most of the enrollees in the student training program were female. Nonetheless, all students in the program had exposure to several BUILD PODER staff, faculty, and students that were from a range of ethnic, gender, and socioeconomic backgrounds. Overall, our findings illustrate that URE programs like BUILD PODER—which seek to help students become aware of and overcome the many science stereotypes that exists—may be key in eliminating gender disparities in science.

### 4.4 | Implications

How can we promote the representation and inclusion of Latinx individuals into biomedical sciences, a group who historically and currently remains less likely to be in the sciences (NSF, 2019)? UREs are an avenue that promote such success (Hernandez et al., 2018). However, we argue that UREs that serve minoritized students should avoid colour-evasive or power-evasive (Neville et al., 2013) programming. Instead, we invite institutions to develop efforts in which programming reflect culturally responsive training that incorporates consideration of minoritized students’ sociocultural identities. We challenge and encourage institutions to include a CRT perspective into their undergraduate research programming efforts (Solórzano et al., 2000), and that both faculty and students be included in this process. This will involve going above and beyond traditional training that is typical of UREs by incorporating a culturally affirming curriculum that (1) makes students aware of power structures within the scientific community, (2) challenges stereotypes and beliefs about who is a scientist (3) discusses the *importance* and *benefits* of culture and various forms of diversity in science, and (4) empowers students by guiding them in recognizing the vast cultural wealth they bring from their families and communities. Engaging in such a process will ensure that Latinx students’ sociocultural identities are understood, uplifted, and empowered across different contexts.

### 4.5 | Limitations and future directions

Our study had several limitations. First, our sample was unique, comprising of one group of Latinx students at a Hispanic-Serving Institution, and thus, may only be interpreted in the context of this specific population investigated. There was also no baseline data available for our constructs under investigation, further limiting our interpretations. Nonetheless, we believe that our results are a foundational step in documenting the powerful nature of how culturally affirming UREs aid in the science identity development of Latinx undergraduate students, a group that pursues science education and careers at disproportionately lower levels (NSF, 2019). In addition, the current research examines *how* identity develops over time. Though fruitful for understanding patterns of science identity development, potential sociocultural factors contributing to variability in these patterns were not thoroughly assessed. Future research should examine *why* such patterns and variability occur (longitudinally). This is important as there is sociocultural variation among Latinx and minoritized students, resulting in several possible pathways in development (Garcia-Cole et al., 1996; Vasquez-Salgado & Greenfield, 2021). As the program continues, future research is slated to examine the science identity development among all minoritized students in BUILD PODER and will also investigate the role of sociocultural factors in identity development, such as first-generation college status.

Furthermore, though our investigation sought to explore science identity, this phenomenon may be interconnected or influenced by other constructs. For example, in reassessing the questions that the program posed to Latinx students (e.g., “*Can you tell us how you are feeling about your skills as a scientist? Do you feel like a scientist? Why or why not?*”), it is quite possible that they tapped into science identity and science self-efficacy. In fact, previous STEM research has examined these two constructs and found them to be closely intertwined, although clearly separable, and have discovered that self-efficacy may be a precursor to identity (Chemers et al., 2011; Robnett et al., 2015; Syed et al., 2019). Nevertheless, the literature has documented that both constructs play a key role in students’ persistence in STEM (Camacho et al., 2021; Chang et al., 2011; Espinosa, 2011). In a separate, but similar vein, students’ conceptualizations of a scientist may have influenced their science identity responses. The conceptualization of what a scientist entails likely varied across the sample and overtime as they navigated engagement in various science activities. Thus, we encourage readers to consider the interconnected nature of these concepts and encourage future URE programs to incorporate measures of science identity and science self-efficacy, as well as what a science person entails, into their programmatic assessments.

Lastly, though BUILD PODER at CSUN is one type of URE, there are multiple URE programs across the nation. We encourage directors of UREs to incorporate similar open-ended questions into their evaluation of students in order to understand the science identity development of minoritized students. Many evaluations of such programming tend to be close-ended and survey-based. It is important to hear students voice, in their own words, how and why they come to feel like a scientist (or not). Additionally, we encourage assessment of non-program students to examine program-specific effects. By incorporating open-ended questions among other UREs as well as non-URE students, this will enable the scientific community to conduct big data analyses that present evidence of the critical role of URE programs in students’ science identity development, and enable us to test the specific role of those that incorporate a culturally affirming curriculum.

## 5 | CONCLUSION

To increase the representation and inclusion of Latinx scientists, institutions must refuse the status-quo of science training and investigate how alternative programming, such as culturally-affirming UREs, can uplift students and their identities to create a more inclusive STEMM. BUILD PODER provides the first empirical evidence of science identity growth among Latinx students through a longitudinal and qualitative perspective.

## Figures and Tables

**TABLE 1:** Qualitative analysis: themes and subthemes

Theme	Subthemes
Science Identity	Achievement (“Scientist”) Moratorium (Unclear) Unexamined (“Not a scientist”)
Science Identity Trajectories to Achievement	Consistent or fast achievement Gradual achievement Achievement adjustment Never reach achievement

**TABLE 2:** Sample demographics and science identity trajectory patterns over the course of 18 months of participation in a CRT-informed undergraduate research experience program

	Science identity					
Trajectory	W1	W2	W3	Gender	Major	Year in Wave 1
Never reach achievement	1	1	1	Male	Life Science, Math or Engineer.	Senior
	1	2	2	Female	Social Science	Sophomore
	2	2	1	Female	Social Science	Junior
	2	2	2	Male	Life Science, Math or Engineer.	Senior
Gradual achievement	1	1	3	Male	Life Science, Math or Engineer.	Junior
	1	2	3	Male	Life Science, Math or Engineer.	Senior
	1	2	3	Male	Social Science	Junior
	2	2	3	Male	Social Science	Junior
	2	2	3	Female	Social Science	Junior
Consistent or fast achievement	3	3	3	Female	Social Science	Junior
	3	3	3	Female	Social Science	Sophomore
	3	3	3	Female	Social Science	Junior
	3	3	3	Female	Life Science, Math or Engineer.	Junior
	2	3	3	Female	Social Science	Senior
	2	3	3	Female	Life Science, Math or Engineer.	Senior
	1	3	3	Female	Life Science, Math or Engineer.	Sophomore
	1	3	3	Female	Social Science	Sophomore
	3	2	3	Female	Life Science, Math or Engineer.	Sophomore
Achievement adjustment	3	3	2	Male	Life Science, Math or Engineer.	Junior
	3	2	2	Male	Social Science	Junior

Note: W1 = Wave 1 (2 weeks); W2 = Wave 2 (6 months); W3 = Wave 3 (18 months); Science Identity Codes (1 = Unexamined [“Not a Scientist”]; 2 = Moratorium [Unclear]; 3 = Achievement [“Scientist”]); Life Science, Math or Engineer. = student is a major within the life sciences (e.g., biology, biochemistry), mathematics or engineering; Social Science = student is a major within the social sciences (e.g., psychology, sociology); Year in Wave 1 = student class standing at their entry into the program.

## Data Availability

As a federally-funded research project, the authors will make available redacted any research materials requested by researchers, including data, coding schemes and analytical procedures.

## Footnotes

1 The status reflects a combination of the *diffused* and *foreclosed* statuses, which prior identity research suggests that may not need differentiation (Phinney, 1989; Syed et al., 2007). Thus, the status ultimately reflects students who do not currently identify as a scientist.
